# Characteristic of Parkinson’s disease with severe COVID-19: a study of 10 cases from Wuhan

**DOI:** 10.1007/s00702-020-02283-y

**Published:** 2021-01-03

**Authors:** Heng Zhai, Yinzhang Lv, Yu Xu, Yi Wu, Weiqi Zeng, Tao Wang, Xuebing Cao, Yan Xu

**Affiliations:** 1 Department of Neurology, Union Hospital, Tongji Medical College, Huazhong University of Science and Technology, Wuhan, 430022 China; 2 Department of Radiology, Tongji Hospital, Tongji Medical College, Huazhong University of Science and Technology, Wuhan, China

**Keywords:** Parkinson’s disease, COVID-19, SARS-CoV-2, Clinical characteristics, Clinical outcomes

## Abstract

Information about Parkinson’s disease (PD) patients with severe COVID-19 is scarce. We aimed to analyze the clinical characteristics, outcomes, and risk factors affecting the prognosis of PD patients with severe COVID-19 infection. Clinical data of severe COVID-19 patients admitted at the Union Hospital, Wuhan between 28th January and 29th February 2020 were collected and analyzed. 10 patients (1.96%) had a medical history of PD with a mean (SD) age of 72.10 (± 11.46) years. The clinical characteristics and outcomes of severe COVID-19 with and without PD patients were then compared. There was no significant difference in overall mortality between the PD and non-PD patients with severe COVID-19 (*p* > 0.05). In PD patients with severe COVID-19, the proportion of patients with critical type, disturbance of consciousness, incidence of complications, white blood cells count and neutrophils counts on admission seem higher in the non-survivors. PD patients with older age, longer PD duration, and late stage PD may be highly susceptible to critical COVID-19 infection and bad outcome. The PD patients with consciousness disorders and complications that progressed rapidly are at increased risk of death.

**Supplementary Information:**

The online version contains supplementary material available at 10.1007/s00702-020-02283-y.

## Introduction

Coronavirus disease 2019 (COVID-19) is an acute infectious respiratory disease caused by severe acute respiratory syndrome coronavirus 2 (SARS-CoV-2). It was first reported in Wuhan, China in December 2019 and quickly spread across the world within few months (Zhu et al. 2020). This virus has poorer outcomes and higher mortality rates in older people and those with comorbidities or chronic diseases. This being the case, patients suffering from Parkinson’s Disease (PD) are also likely to be susceptible to COVID-19 infection and evolve to a severe condition, and possible death, because of their old age and low immunity (Borges et al. 2020; Helmich et al. 2020; Shahid et al. 2020).

To date, there are only few clinical studies on PD patients with active COVID-19 infection. It was showed that PD with older age and longer disease duration are particularly susceptible to COVID-19 with a high mortality (Antonini et al. 2020; Hainque et al. 2020). However, it is not entirely clear whether the mortality of PD is higher than that of the general population. Moreover, the risk factors for death and whether PD itself has a negative effect on COVID-19 patients have not been accurately defined.

The study aims to determine the clinical manifestations, and outcomes of PD patients with severe COVID-19 and to further explore the risk factors associated with in-hospital mortality of PD patients in the early stage of the epidemic. We hope that this work will be valuable for helping clinicians identify patients with poor prognosis at an early stage by catching some of the alarming clinical characteristics presented by PD patients with COVID-19, and guide effective management for PD patients.

As such, clinical data were collected from severe and critically ill COVID-19 patients in West Branch of Union hospital in Wuhan at the peak of COVID-19 outbreak. The clinical characteristics and outcomes of severe COVID-19 with and without PD as well as survivors and non-survivors of PD were then analyzed.

## Methods

### Study design and participants

This was a single-center, retrospective, and observational study. It was approved by the Ethics committee of Tongji Medical College of Huazhong University of Science and Technology. The study also complied with the Declaration of Helsinki (World Medical Association Declaration of Helsinki Ethical Principles for Medical Research Involving Human Subjects 2013). Clinical data of patients positive for COVID-19 in the West Branch of Union Hospital in Wuhan were collected between 28th January and 29th February 2020 for analysis. This was a designated hospital for the severely ill COVID-19 patients. All patients positive for COVID-19 were diagnosed according to the World Health Organization interim guidelines (WHO. Clinical management of severe acute respiratory infection when novel coronavirus (2019-nCoV) infection is suspected: interim guidance, 2020). Oral consent was obtained directly from the patients or the patients’ close relatives prior to data collection.

### Data collection

Demographic data (age, sex, and date of COVID-19 onset) as well as data on the clinical signs and symptoms, medical history, laboratory findings, treatment used, and outcomes were collected from the hospital’s electronic medical records. The date of COVID-19 onset was defined as the day when the symptoms were first noticed. The diagnosis and severity of COVID-19 was determined according to the guidelines outlined in the diagnosis and treatment protocol for novel coronavirus (2019-nCoV) disease (Trial Version 7) compiled by the Chinese National Health Commission. The data were then reviewed by a team of trained physicians. The clinical symptoms and treatment measures of PD patients were also reviewed and confirmed by two trained neurologists.

The treatment measures of COVID-19 patients were collected and included. The patients had at least received antiviral agents, antibiotics, immune regulators, oxygen inhalation and mechanical ventilation. H–Y stage, duration and antiparkinsonian drugs were collected for patients with PD. The length of hospital stays, course of illness and outcome (discharge or death) were also recorded. COVID-19 patients were discharged from the hospital, when they had attained the normal body temperature, remission of clinical symptoms, and resolved lung inflammation proved by a lung computed tomography. The patients displayed at least two consecutive negative results of real-time reverse transcriptase polymerase chain reaction detection for SARS-CoV-2 nucleic acid done at an interval of 24 h.

Laboratory findings on admission including blood cell count, serum alanine aminotransferase (ALT), aspartate aminotransferase (AST), blood urea nitrogen (BUN), creatinine (Cr), creatine kinase (CK), lactate dehydrogenase (LDH), C-reactive protein (CRP), blood glucose, electrolyte, and D-dimer levels were collected.

### Statistical analysis

All statistical analyses were performed using the SPSS software version 23.0. Normality of the data was tested using the Shapiro–Wilk test. Normally distributed continuous variables were presented as means and standard deviations while non-normally distributed continuous variables were presented using median and interquartile range (IQR) values. Categorical variables were expressed as counts and percentages. Means for the normally distributed continuous variables were compared using the independent group *t*-tests while the non-normally distributed data was compared using the Mann–Whitney test. Categorical variables were compared using the unpaired Wilcoxon rank sum test. Their proportions were compared using the *χ*^2^ tests. *p* values less than 0.05 indicated that there were significant differences between groups.

## Results

### Incidence, demographics, and clinical characteristics of PD patients with severe COVID-19 infection

Between 28th January and 29th February 2020, 510 patients were hospitalized at West Branch of Wuhan Union Hospital and confirmed to severe and critically ill COVID-19. The patients had a median age of 59.5 years (IQR 46–68 years). The most common pre-existing comorbidities among the patients were hypertension (32.35%), diabetes (19.77%), cardiovascular diseases (9.59%) and cerebrovascular disease (2.35%). Patients with hypertension and diabetes had a median age of 68 years (IQR 61–74 years) and a mean (SD) age of 65.87 (± 11.86) years; while, those with cardiovascular disease and cerebrovascular disease had a median age of 67 years (IQR 62–76.5 years) and 81 years (IQR 64.25–83.75 years), respectively. Only 10 patients (1.96%) had a medical history of PD. These patients were aged between 56 and 89 years, with a mean (SD) age of 72.10 (± 11.46) years (Tables 1, 3). Data of 286 patients with the same age range and no history of PD were collected to avoid statistical bias caused by age (Table 3). The 286 patients had a median age of 66 years (IQR 62–71 years). There was no significant difference between the age of patients with PD and patients with other comorbidities (*p* > 0.05) (Table 1).

**Table 1:** Comparison in chronic medical illness of COVID-19 patients

	No. (%)	Age (year)	*p* value
Chronic medical illness			
Hypertension	165 (32.35%)	68.00 (61.00–74.00)	0.226
DM	101 (19.77%)	65.87 ± 11.86	0.132
CHD	49 (9.59%)	67.00 (62.00–76.50)	0.422
CVD	12 (2.35%)	81.00 (64.25–83.75)	0.485
PD	10 (1.96%)	72.10 ± 11.46	

*COVID-19* Coronavirus disease 2019, *PD* Parkinson’s disease, *DM* diabetes mellitus, *CVD* cerebrovascular disease, *CHD* coronary heart disease, *No.* number

*p* value indicate differences between age of patients with hypertension, DM, CHD or CVD and patients with PD

*p* < 0.05 was considered statistically significant

The general information, clinical characteristics, treatment, and outcomes of COVID-19 patients with PD are shown in Table 2. All PD patients were either severe or critically ill on admission. Among them, three patients had hypertension, two had diabetes mellitus, four had cerebrovascular disease, and two had cardiovascular disease. The symptoms of PD patients with COVID-19 included coughing (*n* = 10;100.00%), fever (*n* = 8; 80.00%), fatigue (*n* = 7; 70.00%), anorexia (*n* = 6; 60.00%), chest tightness (*n* = 5; 50.00%), shortness of breath (*n* = 4; 40.00%), dyspnea (*n* = 3; 30.00%), myalgia (*n* = 3; 30.00%), diarrhea (*n* = 1; 10.00%) and nausea (*n* = 1; 10.00%) (Table 3). Three patients (30%) had rapid deterioration and died soon after admission.

**Table 2:** Clinical characteristics, treatment and outcomes of COVID-19 patients with PD

No.	Age, year	Gender	H–Y stage	Duration of PD	Medical history				Blood pressure	Severity assessment of COVID-19	Onset time of COVID-19	Comorbidity	Treatment of PD	Treatment of COVID-19	Oxygen inhalation	Length of stays	Outcome	Cause of death
				(year)	HP	DM	CVD	CHD	(mmHg)	Mild/Severe/Critical	(days)		strengths (mg)			(days)		
1	69	F	2	4	No	No	No	Yes	120/75	Severe	1/21/2020	Deep vein thrombosis	Levodopa-Benserazide 200/50 mg 1/2 tablet 3 times daily Pramipexole 0.25 mg 1 tablet 3 times daily Amantadine 100mg 1 tablet once daily	Arbidol Moxifloxacin Thymopeptide Expectorant	Oxygen with nasal cannula	16	Recovery	Nothing
2	56	M	2.5	7	No	No	No	No	120/73	Severe	1/27/2020	Nothing	Levodopa-Benserazide 200/50 mg 1/2 tablet 3 times daily Piribedil 50 mg 1 tablet 3 times daily	Arbidol Moxifloxacin Thymopeptide Expectorant	Oxygen with nasal cannula	24	Recovery	Nothing
3	66	F	1.5	4	No	No	No	No	141/96	Severe	2/6/2020	Nothing	Levodopa-Benserazide 200/50 mg 3/4 tablet 3 times daily Pramipexole CR 0.75 mg 1 tablet once daily	Moxifloxacin Ribavirin Thymopentin	Oxygen with nasal cannula	8	Recovery	Nothing
4	87	F	5	20	No	No	Yes	Yes	120/86	Critical	2/2/2020	Severe hypernatremia	Discontinued	Moxifloxacin Ribavirin Thymopentin	Oxygen with facial mask	6	Died after 6 days of hospitalization	ARDS
5	77	M	5	12	No	Yes	No	No	110/70	Severe	1/15/2020	Nothing	Levodopa-Benserazide 200/50 mg 1/2 tablet 3 times daily	Moxifloxacin Arbidol Expectorant	Oxygen with nasal cannula	21	Transferred to neurorehabilitation	Nothing
6	71	M	4	9	Yes	Yes	Yes	No	141/89	Severe	2/28/2020	Nothing	Levodopa-Benserazide 200/50 mg 1/2 tablet 4 times daily	Arbidol Moxifloxacin	Oxygen with nasal cannula	26	Transferred to neurorehabilitation	Nothing
7	61	F	1.5	2	No	No	No	No	100/64	Severe	1/23/2020	Nothing	Levodopa-Benserazide 200/50 mg 1/2 tablet 3 times daily	Moxifloxacin Arbidol Thymopeptide Expectorant	Oxygen with facial mask	63	Recovery	Nothing
8	62	F	1.5	6	Yes	No	Yes	No	180/102	Critical	2/16/2020	Cerebral hemorrhage	Discontinued	Moxifloxacin Arbidol	Oxygen with facial mask	1	Died within 24 hours	Herniation
9	89	F	5	17	No	No	No	No	151/81	Critical	1/25/2020	Severe anemia, edema, low T3 syndrome	Discontinued	Ceftriaxone Arbidol	Mechanical ventilation	6	Died after 6 days of hospitalization	ARDS
10	83	F	2.5	5	Yes	No	Yes	No	160/79	Severe	2/16/2020	Nothing	Levodopa-Benserazide 200/50 mg 3/4 tablet 3 times daily Pramipexole 0.25 mg 1 tablet 3 times daily	Ribavirin Thymopeptide	Oxygen with facial mask	22	Transferred to neurorehabilitation	Nothing

*F* female, *M* male, *COVID-19* Coronavirus disease 2019, *HP* hypertension, *DM* diabetes mellitus, *CVD* cerebrovascular disease, *CHD* coronary heart disease, *ARDS* acute respiratory distress syndrome

**Table 3:** Demographic, clinical characteristics and outcomes of COVID-19 patients with and without PD

	Total (*n* = 296)	COVID-19 with PD (*n* = 10)	COVID-19 without PD (*n* = 286)	*p* value
Age (year), median (IQR)	66.00 (62.00–71.00)	70.00 (61.75–84.00)	66.00 (62.00–71.00)	0.229
≥ 70 year, No. (%)	106 (35.81%)	5 (50.00%)	101 (35.31%)	0.338
< 70 year, No. (%)	190 (64.19%)	5 (50.00%)	185 (64.69%)	
Gender, No. (%)				
Male	150 (50.68%)	3 (30.00%)	147 (51.40%)	0.213
Female	146 (49.32%)	7 (70.00%)	139 (48.60%)	
Chronic medical illness, No. (%)				
Hypertension	138 (46.62%)	3 (30.00%)	135 (47.20%)	0.349
Diabetes mellitus	81 (27.27%)	2 (20.00%)	79 (27.62%)	0.773
Cerebral vascular disease	12 (3.04%)	4 (40.00%)	8 (2.80%)	0
Malignant neoplasm	23 (7.74%)	0 (0.00%)	23 (8.04%)	1
Heart disease	50 (16.84%)	2 (20.00%)	48 (16.78%)	1
Chronic kidney disease	7 (2.36%)	0 (0.00%)	7 (2.45%)	1
COPD	6 (2.02%)	0 (0.00%)	6 (2.10%)	1
Tuberculosis	2 (0.67%)	0 (0.00%)	2 (0.70%)	1
Severity assessment of COVID-19, No. (%)				
Severe	175 (59.12%)	6 (60.00%)	169 (59.09%)	1
Critical	121 (40.88%)	4 (40.00%)	117 (40.91%)	
Symptoms of COVID-19, No. (%)				
Fever	255 (86.15%)	8 (80.00%)	247 (86.36%)	0.634
Cough	197 (66.55%)	10 (100.00%)	187 (65.38%)	0.034
Fatigue	161 (54.39%)	7 (70.00%)	154 (53.85%)	0.305
Chest tightness	121 (40.88%)	5 (50.00%)	116 (40.56%)	0.745
Shortness of breath	140 (47.30%)	4 (40.00%)	136 (47.55%)	0.753
Dyspnea	102 (34.46%)	3 (30.00%)	99 (34.62%)	1
Myalgia	70 (23.65%)	3 (30.00%)	67 (23.43%)	0.705
Anorexia	61 (20.61%)	6 (60.00%)	55 (19.23%)	0.006
Diarrhea	42 (14.19%)	1 (10.00%)	41 (14.34%)	1
Nausea	25 (8.45%)	1 (10.00%)	24 (8.39%)	0.592
Length of stay (days)	13.00 (7.00–18.75)	12.00 (5.25–24.50)	13.00 (7.00–18.00)	0.804
Duration (days)	26.00 (18.00–31.00)	30.00 (19.00–51.00)	25.00 (18.00–31.00)	0.121
Clinical outcome, No. (%)				
Survived	177 (59.80%)	7 (70.00%)	170 (59.44%)	0.745
Died	119 (40.20%)	3 (30.00%)	116 (40.56%)	

*COVID-19* Coronavirus disease 2019, *PD* Parkinson’s disease, *COPD* chronic obstructive pulmonary disease

*p* value indicate differences between COVID-19 patients with and without PD

*p* < 0.05 was considered statistically significant

COVID-19 patients with and without PD were given the same treatment strategies including antiviral drugs such as abidor, antibiotics, expectorants, and immune regulators such as thymosin. All of the seven remaining PD patients were on antiparkinsonian medication during their hospitalization. Moreover, all of the 10 PD patients required oxygen supplementation; five had a nasal cannula, four had a facial mask and one was under a mechanical ventilation therapy (Table 2). In PD patients with severe COVID-19, the median hospital stay was 12.00 [IQR 5.25–24.50] days, and the median duration of COVID-19 disease was 30 [IQR 19–51] days (Table 3).

### Comparison of clinical characteristics and outcomes between severe COVID-19 patients with and without PD

Table 3 shows the clinical characteristics and outcomes of severe COVID-19 patients with and without PD. This comparison comprised 296 COVID-19 patients (286 without PD and 10 with PD). Majority of the patients were 70 years old or younger. All patients had either severe or critical illness. Among them, 46.62, 27.27, and 16.84% of the patients had hypertension, diabetes, and cardiac disease, respectively. The proportion of patients with cerebrovascular diseases among the PD patients with COVID-19 was 40.00%. This was significantly higher than that of COVID-19 patients without PD (2.8%).

The proportion of PD patients with cough (100.00%, *n* = 10) and anorexia (60.00%, *n* = 6) was significantly higher than that of patients without PD (65.38%, *n* = 187 and 19.23%, *n* = 55, respectively) (*p* < 0.05). There were no significant differences in lengths of hospital stay and duration of disease between patients with and without PD [12.00 (5.25–24.50) vs. 13 (7–18) days and 30 (19–51) vs. 25 (18–31) days, respectively] (*p* > 0.05). The overall mortality of COVID-19 patients aged between 56 and 89 years was 40.20%. In the same line, the mortality was 30.00% and 40.56% in patients with and without PD, respectively. However, this difference was not significant (*p* > 0.05).

### Summary of clinical characteristics and laboratory findings of survivors and non-survivors of PD patients with severe COVID-19

The summary of clinical data of PD patients with severe COVID-19 are shown in Table 4. The median duration of COVID-19 disease of the non-survivors was 13.00 [3.00–21.00] days, and that of the survivors was 43.29 (16.86) days. Further analysis of the disease characteristics of PD patients revealed that seven of them belonged to the non-tremor dominant type. In addition, eight had abnormal mood. Median age of PD survivors and non-survivors was 67.50 years (IQR 62.25–75.50 years) and 87.00 years (IQR 62.00–89.00 years), respectively.

**Table 4:** Clinical characteristics and laboratory findings between survivors and non-survivors with COVID-19 and PD

	COVID-19 with PD			
	Total (*n* = 10)	Survivors (*n* = 7)	Non-survivors (*n* = 3)	
Duration (days)	34.00 ± 20.76	43.29 ± 16.86	13.00 (3.00–21.00)	
Age (year), median (IQR)	70.00 (61.75–84.00)	67.50 (62.25–75.50)	87.00 (62.00–89.00)	
≥ 70 year, No. (%)	5 (50.00%)	3 (42.86%)	2 (66.67%)	
< 70 year, No. (%)	5 (50.00%)	4 (57.14%)	1 (33.33%)	
Gender, No. (%)				
Male	3 (30.00%)	3 (42.86%)	0 (0.00%)	
Female	7 (70.00%)	4 (57.14%)	3 (100.00%)	
Comorbidities, No. (%)				
Hypertension	3 (30.00%)	2 (28.57%)	1 (33.33%)	
Diabetes mellitus	2 (20.00%)	2 (28.57%)	0 (0.00%)	
Cerebrovascular disease	4 (40.00%)	2 (28.57%)	2 (66.67%)	
Coronary heart disease	2 (20.00%)	1 (14.29%)	1 (33.33%)	
Chronic kidney disease	1 (10.00%)	0 (0.00%)	1 (33.33%)	
Characteristics of PD, No. (%)				
Course ≥ 10 years	3 (30.00%)	1 (14.29%)	2 (66.67%)	
5 years ≤ Course < 10 years	4 (40.00%)	3 (42.86%)	1 (33.33%)	
Course < 5 years	3 (30.00%)	3 (42.86%)	0 (0.00%)	
Non tremor dominant	7 (70.00%)	4 (57.14%)	3 (100.00%)	
H–Y grade ≥ 3	4 (40.00%)	2 (28.57%)	2 (66.67%)	
Abnormal mood	8 (80.00%)	5 (71.43%)	3 (100.00%)	
Motor complications	5 (50.00%)	3 (42.86%)	2 (66.67%)	
Cognitive impairment	3 (30.00%)	1 (14.29%)	2 (66.67%)	
Mental disorder	3 (30.00%)	1 (14.29%)	2 (66.67%)	
Dysphagia	3 (30.00%)	1 (14.29%)	2 (66.67%)	
Severity assessment of COVID-19, No. (%)				
Severe	7 (70.00%)	7 (100.00%)	0 (0.00%)	
Critical	3 (30.00%)	0 (0.00%)	3 (100.00%)	
Disturbance of consciousness, No. (%)	4 (40.00%)	1 (14.29%)	3 (100.00%)	
Complication, No. (%)	4 (40.00%)	1 (14.29%)	3 (100.00%)	
Cerebral hemorrhage	1 (10.00%)	0 (0.00%)	1 (33.33%)	
ARDS	2 (20.00%)	0 (0.00%)	2 (66.67%)	
Deep vein thrombosis	1 (10.00%)	1 (14.29%)	0 (0.00%)	
Severe hypernatremia	1 (10.00%)	0 (0.00%)	1 (33.33%)	
Laboratory findings				
White blood cell > 10 × 109/L, No. (%)	4 (40.00%)	1 (14.29%)	3 (100.00%)	
Neutrophils > 9 × 109/L, No. (%)	4 (40.00%)	1 (14.29%)	3 (100.00%)	
Lymphocyte < 1 × 109/L, No. (%)	6 (60.00%)	3 (42.86%)	3 (100.00%)	
Hemoglobin < 90 g/L, No. (%)	1 (10.00%)	0 (0.00%)	1 (33.33%)	
Blood platelet < 125 × 109/L, No. (%)	2 (20.00%)	0 (0.00%)	2 (66.67%)	
C-reactive protein > 8 mg/L, No. (%)	8 (80.00%)	6 (85.71%)	2 (66.67%)	
Serum sodium > 145 mmol/L, No. (%)	3 (30.00%)	1 (14.29%)	2 (66.67%)	
Blood glucose > 6.1 mmol/L, No. (%)	7 (70.00%)	4 (57.14%)	3 (100.00%)	
D-dimer > 0.5 mg/L, No. (%)	8 (80.00%)	5 (71.43%)	3 (100.00%)	
Alanine aminotransferase, U/L		23.00 (10.00–52.00)	17.00 (4.00–50.00)	
Aspartate aminotransferase, U/L		42.29 ± 30.83	34.00 (9.00–38.00)	
Blood urea nitrogen, mmol/L		6.97 (3.53–7.95)	6.36 (5.62–16.57)	
Creatinine, μmol/L		56.97 ± 17.01	82.1 (53.8–93.4)	
Creatine kinase, U/L		93.00 (68.00–156.25)	124.00 (22.00–426.00)	
Lactate dehydrogenase, U/L		292.29 ± 118.71	265.00 (181.00–407.00)	
Antiparkinsonian drug treatment, No. (%)				
Levodopa	7 (70.00%)	7 (100.00%)	0 (0.00%)	
Dopamine receptor agonist	4 (40.00%)	4 (57.14%)	0 (0.00%)	
Amantadine	1 (10.00%)	1 (14.29%)	0 (0.00%)	
Discontinuation of antiparkinsonian drug	3 (30.00%)	0 (0.00%)	3 (100.00%)	

*COVID-19* Coronavirus disease 2019, *PD* Parkinson’s disease, *NA* not available, *ARDS* acute respiratory distress syndrome

In chronic comorbidities, 28.57% of the survivors and 66.7% of the non-survivors have history of cerebrovascular disease. Based on the Hoehn and Yahr scale (H&Y), 28% of the survivors and 67% of the non-survivors were in H&Y stage > 3, and two of three non-survivors was in H&Y stage 5. Among the survivors, only one patient (14%) had duration of PD over ten years, while two (66%) of three non-survivors had more than ten years of PD course. One (14%) of the survivors and two (66%) of non-survivors had cognitive impairment, mental disorder and dysphagia. The three non-survivors were all critical illness. The proportion of patients with disturbance of consciousness on admission in the non-survivors (100%) seemed higher than that of the survivors (14.29%). Similarly, the non-survivors seemed to have a higher white blood cell count and neutrophil count.

Moreover, all of the non-survivors and one of survivors had incidence of complications. Among three PD patients (30%) who died, one 62-year-old patient died from cerebral hernia that was secondary to cerebral hemorrhage within 24 h after admission. The other two non-survivors who were aged above 85 years died from ARDS that rapidly deteriorated into severe complications, with one non-survivors had severe hypernatremia, while the other had severe edema. Only one patient among the seven survivors developed venous thrombosis of lower extremity.

Regarding the antiparkinsonian drugs applied during hospitalization, the three non-survivors did not receive antiparkinsonian drugs anymore, and the seven survivors continued using antiparkinsonian drugs. Levodopa was used by all survivors.

## Discussion

This was a single-centered retrospective study that described the incidence, clinical characteristics, outcomes and relative risk factors of PD patients with either severe or critical COVID-19. Early diagnosis and timely treatment to reduce mortality are of crucial importance. PD patients may gradually worsen the motor or non-motor symptoms in the early stages of COVID-19 (Hainque et al. 2020). In our study, the proportion of PD patients with anorexia was higher than those without PD. The mechanism of appetite regulation among PD patients is complex. It involves a decrease in anorexigenic hormones and dopaminergic reward mechanisms, which in turn leads to anhedonia and influences food intake habits (Cersosimo et al. 2018; Der-Avakian et al. 2012). SARS-CoV-2 was found to enter the brain via olfactory bulbs in a mouse model. This explained the potential pathogenic mechanism of taste and smell disorders in patients with COVID-19 infection (Netland et al. 2008; Xu et al. 2020). Early and accurate diagnosis of COVID-19 in PD patients may be challenging (Hainque et al. 2020). As such, the focus should be given to poor appetite in PD patients during the COVID-19 epidemic.

Common comorbidities among patients with COVID-19 with and without PD were hypertension, diabetes mellitus, and coronary heart disease. This was consistent with those reported by Huang and colleagues (Huang et al. 2020). However, the proportion of patients with cerebrovascular disease was higher among those with PD than among those without PD, and the PD non-survivors seemed have higher incidence of cerebrovascular disease. Previous studies found that patients with stroke were more likely to have PD, and PD increases stroke risk and influences post-stroke outcomes (Huang et al. 2019). SARS-CoV patients with cerebrovascular disease have a higher risk of critical illness and multiple organ dysfunction (Aggarwal et al. 2020). It was speculated that patients with PD and cerebrovascular disease were more susceptible to become severely ill with COVID-19 and progress to death.

Studies on COVID-19 patients requiring services of the intensive care unit (ICU) revealed that their mortality was 61.5% (Yang et al. 2020a). Our study was conducted in a hospital designated for severely ill COVID-19 patients who were hospitalized with hypoxemia, and thus, included those requiring ICU services and those who did not. The mortality rate of PD patients was 30.00%, while that of non-PD patients was 40.20%. In our study, the high rate of mortality rate of severe COVID-19 patients with PD was in the early stage of the epidemic, and it needs a larger sample size to be confirmed.

Whether various characteristics of PD have different effects on COVID-19 patients is not entirely clear. A community-based case–control study revealed that mild-to-moderate COVID-19 occurred independent of age and PD duration (Cilia et al. 2020). Another study indicated that COVID-19 risk, morbidity and mortality in patients with mild-to-moderate PD did not differ from the general population (Fasano et al. 2020). Antonini (Antonini et al. 2020) examined 10 PD patients drawn from two Parkinson Units in Europe and reported that advanced PD patients had a higher COVID-19 mortality rate, which is associated with older age and longer disease duration. In our study, those PD patients who had older age, H–Y stage > 3 and disease course > 10 years seem more likely to progress to death once infected with severe COVID-19. This conclusion needs to be further confirmed by large sample control studies.

In our study, non-survivors were critically ill and had loss of consciousness on admission. These patients exhibited rapid deterioration than the survivors, which was similar to the previous study that patients in a more critical condition at ICU admission are at increased risk of death. The most common cause of death was acute respiratory distress syndrome (ARDS), and most patients revealed organ function damage in critically ill patients with COVID-19(Yang et al. 2020b).

A total of four PD patients had incidence of complications, three non-survivors died of complications within a week. Two of them died of ARDS. One died of cerebral hemorrhage within 24 h post admission, and suffered a cerebral infarction few years before the pandemic. Nevertheless, a survivor with deep vein thrombosis of the lower extremity and without a history of cerebral infarction recovered and got discharged after anticoagulant treatment. Patients with COVID-19 are prone to develop coagulation disorders. The imbalance of platelet production and destruction as well as the disorder of the coagulation system increased the risk of bleeding in patients with COVID-19 (Tang et al. 2020). Moreover, the risk of thrombosis was higher in patients with COVID-19 (Li et al. 2020). Herein, 8 PD patients demonstrated increased D-dimer levels. Though no thrombus was found in two patients who died of ARDS, dynamic analysis revealed that one had a gradual increase in D-dimer levels, a gradual decrease in platelet and hemoglobin levels, and severe hypernatremia during the short course of the COVID-19 disease, but had no abnormality during admission (Supplementary Table). In some patients, especially in severe and dead patients, the platelet levels are significantly reduced (Guang et al. 2020; Tang et al. 2020; Wang et al. 2020). Hypernatremia is associated with a hypercoagulable state particularly in an immobile patient, and may be common in COVID-19 patients in ICU (Christ-Crain et al. 2020). Thus, platelet count, D-dimer levels, hemoglobin levels and electrolyte levels at the beginning of admission cannot be used as a guide of severity. A single-center cohort study in Japan showed that diffusive intravascular coagulation (DIC) score may be common clinical predictor of mortality with ARDS (Anan et al. 2018). We speculate that the delayed but aggravated coagulation disorders may mediate the fatal ARDS resulting in death of the PD patients with severe COVID-19. Therefore, these laboratory indicators need to be closely monitored after admission to find coagulation disorders as soon as possible, and an anticoagulant was, therefore, administered in time to avoid the disorder.

Neutrophilia and lymphocytopenia were common findings and more significant among severe cases when compared to mild cases with COVID-19 (Chen et al. 2020; Gu et al. 2005; Mo et al. 2020). In our study, lymphocytopenia occurred in 42.86% survivors and 100% non-survivors with PD, which is in accordance with the previous studies, suggesting that the severity of lymphocytopenia reflects the severity of SARS-CoV-2 infection. Similarly, the white blood cell count and neutrophil count seem higher in the non-survivors in our study, suggesting the indicators are associated with the severity of illness and thus prognosis of patients with PD and severe COVID-19. In addition, our results showed that two non-survivors who died of ARDS presented a gradual decrease in lymphocyte count with a gradual increase in neutrophil count and the survivors lacked these dynamic changes during the course of the COVID-19 disease (Figure s1, Figure s2), which was consistent with the statement that peripheral neutrophilia and lymphocytopenia associated with delayed but exaggerated immune response mediate the fatal ARDS, multi-organ dysfunction resulting in death of COVID-19 patients (Lingeswaran et al. 2020). Hence, dynamic neutrophilia and lymphocytopenia may help in early diagnosis and predicting severity of PD patients with COVID-19.

Sudden cessation of antiparkinsonian drugs leads to aggravation of PD symptoms and even cause malignant syndrome (Grover et al. 2018). In older advanced PD patients with pre-existing dyspnea, it causes rigidity of the respiratory muscle and impairment of the cough reflex thereby leading to increased severity of COVID-19 and death (van Wamelen et al. 2020). Study of the Kings’ College group showed that most PD patients required additional levodopa dosing following the infection (Antonini et al. 2020). Another study demonstrated that motor and non-motor symptoms significantly worsened in the COVID-19 group, requiring therapy adjustment in one-third of cases (Cilia et al. 2020). In our study, three PD patients who died did not have symptoms of advanced malignant syndrome such as high fever, rigidity, tremors, and significant elevation of creatine kinase. We speculate that withdrawal of antiparkinsonian drugs in 3 non-survivors may be due to loss of consciousness or loss of the chance to take medicine. Withdrawal of parkinsonian drugs aggravates the symptoms of PD and promotes death. This, however, needs to be studied in larger clinical studies.

Nevertheless, this study was limited by several factors. It was conducted at a single-center hospital and thus the interpretation of the findings could have been limited by the small number of PD patients. We speculate that the low proportion of COVID-19 patients with PD may be due to lack of outdoor exercise and less social contact with others in patients with PD during the periods of COVID-19 prevalence. In the same line, the effect of COVID-19 on the PD symptoms was not analyzed, because the relevant information was not collected. Moreover, the impact of COVID-19 on PD was not evaluated for long periods of time. As such, the impact of COVID-19 on PD needs to be evaluated with long-term follow-up of survivors.

## Conclusion

PD patients with the older age, longer PD duration and later stage PD seem have a worse prognosis after getting severe COVID-19. COVID-19 severity and presence of complications could greatly affect the prognosis of the PD patients with severe COVID-19. It is necessary to pay special attention to the loss of appetite of PD patients in addition with other COVID-19 symptoms for early and accurate diagnosis of COVID-19. Dynamic monitoring of organ function and coagulation indicator is needed to identify these changes early with prompt management and prevent further complications. Sustainable and optimized antiparkinsonian therapy may be needed to improve the prognosis of PD patients with COVID-19.

## Supplementary Information

Below is the link to the electronic supplementary material.

Supplementary file1 (DOCX 55 KB)

Supplementary file2 (DOCX 55 KB)

Supplementary file3 (DOCX 15 KB)

## Data Availability

The data that support the findings of this study are available from the corresponding author upon reasonable request.

## Abbreviations

PD: Parkinson’s disease
COVID-19: Coronavirus disease 2019
SARS-CoV-2: Severe acute respiratory syndrome coronavirus 2
ALT: Serum alanine aminotransferase
AST: Aspartate aminotransferase
BUN: Blood urea nitrogen
Cr: Creatinine
CK: Creatine kinase
LDH: Lactate dehydrogenase
CRP: C-reactive protein
ICU: Intensive care unit
ARDS: Acute respiratory distress syndrome
DIC: Diffusive intravascular coagulation
