# Supplementary material for: Characteristic of Parkinson’s disease with severe COVID-19: a study of 10 cases from Wuhan
Source: J Neural Transm (Vienna). 2021 Jan 3;128(1):37–48. doi: 10.1007/s00702-020-02283-y (PMC7779096; doi:10.1007/s00702-020-02283-y)
Supplement: Supplementary file 1 — Supplementary file1 (DOCX 55 KB) [file 702_2020_2283_MOESM1_ESM.docx]

Figure s1: Tendency chart of neutrophil counts in PD patients during hospitalization
