# Supplementary material for: Characteristic of Parkinson’s disease with severe COVID-19: a study of 10 cases from Wuhan
Source: J Neural Transm (Vienna). 2021 Jan 3;128(1):37–48. doi: 10.1007/s00702-020-02283-y (PMC7779096; doi:10.1007/s00702-020-02283-y)
Supplement: Supplementary file 2 — Supplementary file2 (DOCX 55 KB) [file 702_2020_2283_MOESM2_ESM.docx]

Figure s2: Tendency chart of lymphocyte counts in PD patients during hospitalization
