# Supplementary material for: Characteristic of Parkinson’s disease with severe COVID-19: a study of 10 cases from Wuhan
Source: J Neural Transm (Vienna). 2021 Jan 3;128(1):37–48. doi: 10.1007/s00702-020-02283-y (PMC7779096; doi:10.1007/s00702-020-02283-y)
Supplement: Supplementary file 3 — Supplementary file3 (DOCX 15 KB) [file 702_2020_2283_MOESM3_ESM.docx]

| **Supplementary Table: Dynamic laboratory test results of Patient 4 in PD group** | | | | |
| --- | --- | --- | --- | --- |
|  |  | Number of tests | |  |
| Laboratory findings | | First | Second |  |
| Red blood cell, ×10^12^/L | | 3.34 | 2.51 |  |
| Blood platelet, ×10^9^/L | | 177 | 113 |  |
| Serum sodium, mmol/L | | 148.7 | 160.5 |  |
| D-dimer, mg/L | | 0.38 | 3.06 |  |
|  |  |  |  |  |
